# Using Force Plates to Monitor the Recovery of Vertical Jump Performance After Strenuous Exercise: A Systematic Review and Meta-Analysis

**DOI:** 10.3390/jfmk10020230

**Published:** 2025-06-18

**Authors:** Caden Williams, Katherine Sullivan, Changki Kim, Lee J. Winchester, Michael V. Fedewa

**Affiliations:** 1 Department of Kinesiology, The University of Alabama, Tuscaloosa, AL 35487, USA; caden.williams@uga.edu (C.W.); ckim23@ua.edu (C.K.); ljwinchester@ua.edu (L.J.W.); 2 Division of Kinesiology, Health, and Sport Studies, Wayne State University, Detroit, MI 48202, USA; hr2070@wayne.edu

**Keywords:** neuromuscular fatigue, recovery, vertical jump, systematic review, meta-analysis

## Abstract

**Background:** Force plates are commonly used as a non-fatiguing measure of recovery. However, the recovery time course captured via the force plate assessment of vertical jumps has yet to be established. Therefore, the objective of this systematic review and meta-analysis was to examine the change in vertical jump performance and the time course of recovery following an acute bout of strenuous exercise using force plates. **Methods:** Peer-reviewed articles (n = 22) published prior to 8 November 2023, were identified by searching three electronic databases (PubMed, Scopus, Web of Science). Studies included in this review met the following criteria: (1) available in English; (2) involved adult participants >18 years of age; (3) measured the change in vertical jump performance over consecutive days using a force plate system. Individual effect sizes (ESs) were calculated by dividing the change in vertical jump at each timepoint (24 h, 48 h, etc.) by the pooled standard deviation (SD), and they were aggregated using a three-level random-effects model. **Results:** Vertical jump performance decreased following an acute strenuous exercise bout (ES = −0.2639; *p* < 0.0001) and returned to baseline after 3 days of recovery, with larger decreases observed when assessed using Peak Height rather than Peak Power (ES = −0.4687 vs. ES = −0.1399; *p* = 0.0393). Older participants showed a larger decrease in vertical jump (β = −0.0489; *p* < 0.0001). **Conclusions:** Force plates can be used to evaluate recovery post-exercise, with a decline in performance on Days 1 and 2, and full recovery by Day 3. The findings from this study support the use of force plates for the evaluation of recovery.

## 1. Introduction

The demanding nature of athletic activity has been shown to induce substantial fatigue across various sports [1]. Therefore, proper recovery after intense exercise or sport-specific training is essential for maintaining overall health and enhancing athletic performance. This recovery period allows the body to repair skeletal muscle and replenish energy stores and reduces the risk of musculoskeletal injury [2]. Without adequate recovery, individuals may experience overtraining, which can lead to fatigue, decreased performance, and a higher likelihood of injury [3]. Overtraining syndrome (OTS) is a condition characterized by a decline in physical performance and a range of physiological and psychological symptoms resulting from excessive training and insufficient recovery time [4]. A sufficient rest and recovery period is vital to the prevention of OTS and to optimizing muscular recovery [5].

Accurate measurements that quantify performance changes following a fatiguing stimulus, or an injury, are imperative to ensure individual safety, optimal recovery, and readiness to perform. Rapid field-based measurements, such as perceptual recovery, have been validated but can be subjective and have the potential to be influenced by numerous confounding variables [6]. Various non-fatiguing performance measures are used to objectively measure recovery as well, with the squat jump (SJ), countermovement jump (CMJ), and drop jump (DJ) used most commonly by practitioners [7,8]. When compared to other methods (e.g., contact mats, video analysis, photoelectric sensors), force plate systems may provide additional performance metrics beyond maximum jump height to more completely characterize the recovery status of an individual by comparing performance to a known baseline value [9,10,11]. For example, the execution of tasks such as the CMJ is dependent on several time and force components exhibited in the phases preceding the onset of the flight phase [12]. In this context, force plate-derived metrics—such as the flight time-to-contraction time ratio (FT/CT) and the modified reactive strength index (RSImod)—may provide additional insights for fatigue and recovery monitoring [12]. This is possible thanks to the capability of force plates to measure ground reaction forces in three dimensions, which provides valuable information to more completely characterize the lower body movement and assess various biomechanical parameters to help monitor training progression and recovery [13].

In summary, force plate systems are frequently found in clinical and athletic settings and have numerous means of estimating recovery. Evaluation of Peak Power during a force plate jump assessment quantifies the amount of force and speed produced during takeoff, providing data on the explosiveness of a movement [14]. Peak jump height is another frequently used metric that uses flight time to estimate changes in performance, assuming that greater muscular strength and power is positively associated with jumping performance [14]. Flight time (i.e., time spent from takeoff to landing during a jump) allows for the estimation of jump height based on individual anthropometrics. A decrease in these measurements from a baseline assessment would likely indicate a drop in performance due to fatigue or insufficient muscular recovery.

The force plate systems can accurately monitor recovery by precisely measuring data and providing immediate, detailed, and objective feedback related to performance, neuromuscular fatigue, and readiness for a “return to play” [15]. If a decrease in performance is observed, practitioners could then implement the most appropriate recovery strategy [16]. By providing real-time feedback, force plates allow coaches, trainers, and other practitioners to make immediate adjustments to an individual’s recovery plan. However, the specific time-course for determining recovery using force plate metrics have yet to be established. Therefore, the aim of this systematic review and meta-analysis was to examine the change in vertical jump performance and time course of recovery following an acute strenuous exercise bout using force plates.

## 2. Methods

**Figure 1:**
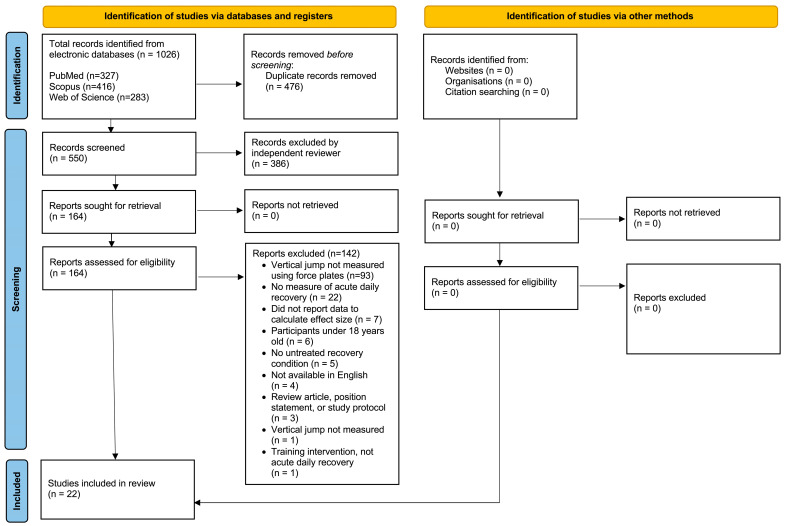
Flow chart of study selection.

This review was conducted in accordance with the PRISMA (Preferred Reporting Items for Systematic Reviews and Meta-analyses) statement guidelines [17], with the corresponding study protocol publicly available to review (ResearchRegistry.com, Protocol ID reviewregistry1994). Articles published prior to 8th November 2023 were located by searching three electronic databases using combinations of the terms “countermovement jump”, “fatigue”, and “recovery” [18]. Databases were searched from inception. A flowchart depicting study selection is provided in Figure 1, and the total numbers of initial articles retrieved and search strategy used for each database were as follows: PubMed (n = 327; “countermovement jump”[All Fields] AND (“fatiguability”[All Fields] OR “fatiguable”[All Fields] OR “fatigue”[MeSH Terms] OR “fatigue”[All Fields] OR “fatigued”[All Fields] OR “fatigues”[All Fields] OR “fatiguing”[All Fields] OR “fatigueability”[All Fields]) AND (“recoveries”[All Fields] OR “recovery”[All Fields])), Scopus (n = 416; (TITLE-ABS-KEY (“countermovement jump”) AND TITLE-ABS-KEY (fatigue) AND TITLE-ABS-KEY (recovery))), and Web of Science (n = 283; Results for “countermovement jump” (All Fields) AND fatigue (All Fields) AND recovery (All Fields)).

### 2.1. Study Selection and Quality Assessment

The titles, abstracts, and full-text articles of potential studies were independently reviewed by two authors (CW and MVF), with disagreements resolved by consulting a third reviewer (LJW). Strict inclusion criteria were used to include articles in the final analysis, and all eligible studies (1) were peer-reviewed publications, (2) were available in English, (3) involved adult participants >18 years of age, (4) measured vertical jump performance using a force plate system, (5) monitored the change vertical jump performance as a recovery measure over consecutive days without the use of recovery aids (i.e., dietary supplement, cold water immersion, compression garments), and (6) reported results as means and standard deviations, standard errors, or 95% confidence intervals from which an effect size could be calculated. Additional authors were contacted for missing or incomplete data not presented in the original research study but did not respond or were unable to provide the requested information [19,20,21,22,23,24].

Methodological study quality was also assessed, independently, by two researchers (KS and MVF) using the NIH Quality Assessment Tool for Before–After (Pre–Post) Studies with No Control Group (Supplemental Table S1) [25]. Quality assessment scoring was conducted as follows: One point was awarded for each appraisal criterion that was met. The sum of all appraisal points was calculated for each article and used such that studies with less than 50% of study quality items satisfied were considered of low methodological quality, studies with 50–75% of study quality items satisfied were considered of moderate methodological quality, and studies with over 75% of study quality items satisfied were considered of high methodological quality.

### 2.2. Data Extraction Strategy

Two researchers (CW and MVF) independently extracted and included the data in a standardized spreadsheet. Sample characteristics including age, sex, body mass, body mass index, height, athlete status, and exercise stressor were all extracted for descriptive characteristics and potential independent variables. The following information was also extracted: The first author and year of publication were recorded, as well as the primary outcome measure (vertical jump performance) for each group of participants at each measurement timepoint. Only data from control groups were extracted from intervention studies. Recovery timepoints were extracted in hours and adjusted to 5 categories according to their respective timepoint ranges: Day 1 (1–24 h), Day 2 (25–48 h), Day 3 (49–72 h), Day 4 (73–96 h), and Day 5 (97–120 h). Data were extracted from graphs [26,27,28,29,30,31,32,33] using the ruler measurement tool and XY coordinates in Adobe Acrobat (v2025.001.20476, San Jose, CA, USA) [34]. Additional descriptions of potential moderators are found in Table 1.

### 2.3. Statistical Analysis

Each ES was calculated by dividing the difference between the mean baseline or pretest vertical jump performance value and the vertical jump performance value at each of subsequent timepoint (24 h, 48 h, 72 h, etc.) by the pooled standard deviation (SD) values [35,36]. A decrease in vertical jump performance resulted in a negative ES. A standardized mean difference effect size (ES) was chosen to quantify the changes in vertical jump performance because results could be reported in different units of measure (i.e., seconds, watts, Newtons, meters, meters/second). Each ES was qualitatively described as small, medium or large (ES = 0.2, 0.5, and 0.8, respectively) [37]. Random-effects models were used to aggregate a mean ES and 95% confidence interval using a 3-level meta-analysis model to account for (1) multiple primary outcome measures of vertical jump performance, (2) multiple timepoints within a single group of participants, and (3) multiple groups of participants within a single study. This approach was necessary, as multiple effects were gathered from studies which stratified results by athlete type (e.g., power, strength, or endurance) or sex and studies involving repeated measures across consecutive days. This model allowed us to adjust for between-study variance and the correlation between effects nested within studies [38,39]. Individual effects were weighted by the inverse variance and aggregated using restricted maximum-likelihood estimation. Data were independently extracted by two authors (CW and MVF), with discrepancies resolved by a third reviewer (LJW) prior to aggregating effects.

Heterogeneity was indicated if the Q total reached a significance level of *p* < 0.05 and was assessed by the examination of the *I^2^* statistic. An *I^2^* value was categorized as low, moderate, or high based on calculations equal to 25%, 50%, or 75%, respectively [40]. In the event of significant heterogeneity, an attempt was made to explain the observed heterogeneity using a similar 3-level meta-regression analysis with robust variance estimation based on several independent variables chosen a priori due to their influence on fatigue and recovery, including age [41,42], athlete type [1,43], exercise stressor [1], and sex [44,45]. A complete list of independent variables can be found in Table 1. The association between these independent variables and fatigue and recovery are reported as unstandardized beta coefficients (β) in the exploratory moderator and subgroup analysis. 

Potential bias was addressed using Egger’s test [46] and assessed using a funnel plot to identify potential asymmetry and outliers. If potential bias was observed, a sensitivity analysis was performed by removing potential outlier effects and reanalyzing the results. In addition, the number of unpublished or unretrieved null effects that would diminish the significance of observed effects to non-significant results was estimated using a fail-safe N [47]. A fail-safe N is often considered robust when the estimated value exceeds 5*k* + 10, in which *k* represents the number of original effects, and estimates when potential bias can be “safely ignored” [48]. All statistical analyses were performed using the “metafor” package in R (v 4.2.1) [49]. Data available for study and participant characteristics are presented as M ± SD unless otherwise indicated.

## 3. Results

A total of 1026 articles were returned with the initial search process, yielding 550 sources after duplicates were removed (Figure 1). Of these, 386 articles were excluded after screening titles and abstracts. The full texts of 164 articles were reviewed, and 142 were excluded after not meeting the eligibility criteria. While the electronic databases were searched from inception, no eligible studies were published before 2000, which yielded a total of 22 studies published between 2000 and 2023 that met the inclusion criteria. A manual review of the article references was conducted to identify additional publications not discovered by the database search [50,51]; however, no additional publications resulted from the manual search of references.

The cumulative results from 296 participants (*k* = 184 individual effects) indicated that vertical jump performance decreased following an acute strenuous exercise bout (ES = −0.2639; 95% CI: −0.3848 to −0.1431; *p* < 0.0001) as indicated by lower Peak Height, Peak Power, Velocity, Flight Time, and Maximum Force, as well as an increase in Total, Concentric, and Eccentric Duration and Vertical Stiffness (among others) (Supplemental Figure S1A–D) [26,27,28,29,30,31,32,33,52,53,54,55,56,57,58,59,60,61,62,63,64,65]. The majority of effects (*k* = 131 effects, 71.2%) were less than zero, with observed effects ranging from −4.5734 to 1.2320. While some characteristics were inconsistently reported, participants ranged in age from 18 to 45 and were predominantly men (n = 266, 89.9%), with only one study which included female participants [56] and two studies consisting of a mixed sample [33,57]. Additional participant and study characteristics can be found in Table 2 and Table 3.

### 3.1. Exploratory Moderator and Subgroup Analysis

When examining the time course of recovery, the number of days following the acute exercise bout was positively associated with the change in vertical jump performance (Table 4). The current results indicated that the decrease in performance became smaller as the number of days since the exercise bout increased. After the strenuous exercise bout occurred on Day 0, vertical jump performance decreased on Day 1 (*k =* 78; ES = −0.4186; 95% CI: −0.5749 to –0.2624; *p* < 0.0001) and Day 2 (*k =* 59; ES = −0.1974; 95% CI: −0.3544 to –0.0403; *p* = 0.0147). However, performance returned to baseline on Day 3 (*k =* 31*;* ES = −0.0966; 95% CI: −0.2443 to 0.0512; *p* = 0.1920) and remained recovered beyond Day 4 (*k =* 16*;* ES = −0.0061; 95% CI: −0.2047 to 0.1926; *p* = 0.9489).

Interestingly, despite yielding a decrease in performance nearly half the size, the decrease in Vertical Jump Performance was not different between groups when comparing athletes to non-athletes (Table 5). In addition, the interaction of Day×Athlete was not significant (β = 0.0560; 95%CI −0.0439 to 0.1559; *p* = 0.2700), which collectively indicated that the time course of recovery was similar between athletes and non-athletes.

The two most commonly reported Primary Outcome of vertical jump performance yielded slightly different results, as the effects using Peak Height showed a larger decrease in performance than the effects using Peak Power (Table 5). Primary Outcome (Peak Height versus Peak Power) and *Day* were both independently associated with the change in performance, such that Peak Height showed a larger decrease in performance (*p* = 0.0296) even after controlling for the number of recovery days (*p* = 0.0008). However, the interaction of Day × Primary Outcome was not significant (β = −0.0333; 95%CI −0.1727 to 0.1060; *p* = 0.6362), which indicated that the time course of recovery measured using Peak Height was not different than when measured using Peak Power.

In addition to Peak Height and Peak Power, other novel measures of performance were occasionally reported such as velocity [26], vertical stiffness [52,55], maximum force and energy production [56], and a number of time and rate-related variables including total, concentric, eccentric, and stretching phase duration, the rate of force development, and the FT/CT ratio, among others [65]. However, because of the relatively low number of studies assessing these additional variables, these effects could not be separately analyzed to determine if one provided a more sensitive gauge of fatigue and recovery. In some cases these time- and rate-related variables (specifically the stretching phase rate of force development (StrRFD), average rate of force development (avgRFD), and total time to peak force (TTPF)) showed a decrease in performance nearly twice as large and remained lower nearly twice as long as the force-based variables (e.g., Peak Force, Peak Power) post-exercise [65]. In fact, the FT/CT ratio was the only measure that was sensitive enough to capture the decrease in performance following a high-intensity exercise bout in a sample of 21 male athletes, with no changes observed when using Peak Height or Peak Power. Vertical stiffness (Kvert) was another novel outcome reported as a measure of the stiffness of the lower body during the contact phase of the jump [52,55], but with somewhat inconsistent results. A slight increase in Kvert was observed 24 h after concentric cycling, with a small decrease observed 24 h after eccentric cycling [52]. In contrast, Kvert showed a slight increase immediately after a single resistance training session before returning to baseline at 24-, 48- and 72 h. However, these changes were non-significant and were smaller than the decrease in performance observed in Peak Height and Peak Power [55]. The magnitude and time course of changes in Kvert, as well as the relative utility of Kvert as a measure of fatigue and recovery compared to other force plate metrics, remain unclear because of these inconsistent responses to different exercise stressors.

Age was inversely associated with change in vertical jump performance, which indicated that older participants showed a larger decrease in vertical jump following an exhaustive exercise bout (Table 4). Interestingly, the decrease in vertical jump performance following a laboratory-based exhaustive exercise bout was not different than the decrease in vertical jump performance following a game or simulated game-play bout (Table 5). The Exercise Stressor × Primary Outcome (β = −0.1573; 95% CI −0.6950 to 0.3804; *p* = 0.5632), Athlete Status × Primary Outcome (β = −0.1135; 95%CI −0.5008 to 0.2737; *p* = 0.5632), and Age × Primary Outcome (β = −0.0099; 95%CI −0.1251 to 0.1053; *p* = 0.8646) interactions were not associated with the decrease in vertical jump. Only the Sex × Primary Outcome (β = 2.5884; 95%CI 1.7051 to 3.4716; *p* < 0.0001) interaction was significantly associated, which indicated that studies with female participants measuring performance using Peak Power showed larger decrease in vertical jump. Additional exploratory moderator analysis and subgroup comparisons can be found in Table 4 and Table 5, respectively.

### 3.2. Assessment of Bias and Sensitivity Analysis

**Figure 2:**
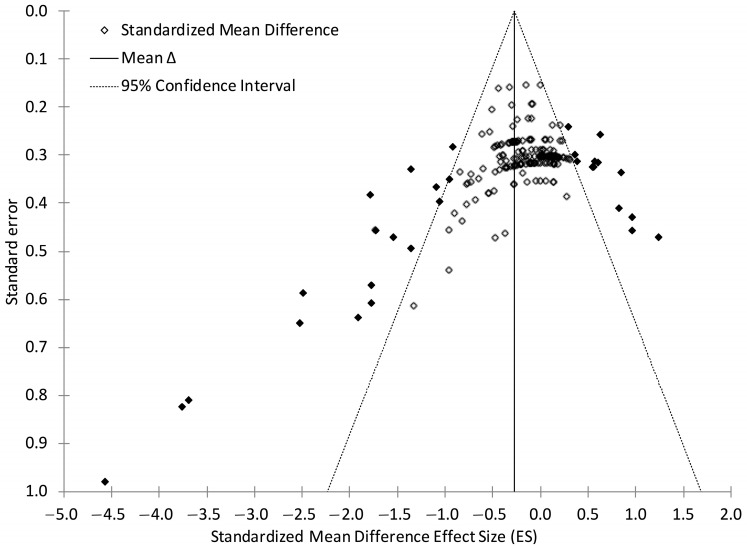
Funnel plot of effect size versus study standard error. Black diamonds are used to indicate potential outliers. White diamonds represent effects within the 95% confidence interval not considered to be potential outliers.

The regression model indicated that the funnel plot (Figure 2) was asymmetrical and was subject to potential bias (β = 0.8004; *p* < 0.0001). After visually inspecting the funnel plot, a sensitivity analysis was performed, removing 26 effects as potential outliers outside of the 95% confidence interval. Removing these effects slightly decreased the mean effect (ES = −0.1930; 95% CI: −0.2685 to −0.1175; *p* < 0.0001) for the remaining 158 effects but eliminated the observed heterogeneity (Q_157_ = 114.8471; *p* = 0.9953).

The fail-safe N, representing the number of unpublished or unretrieved null effects that would diminish the significance of observed effects to non-significant results, was calculated with potential outliers included (n = 5503 effects) and again with potential outliers removed (n = 1920 effects) [47] and indicated that potential bias can be “safely ignored” [48]. In addition, the decrease in vertical jump performance observed in the current analysis likely represents the true change following an exhaustive exercise bout and is not a result of a few extreme values influencing the overall mean ES.

Lastly, each of the studies included in this review were of high methodological quality, with scores ranging from 8 to 12 out of 12 possible points awarded (Table 6). Overall study quality was not associated with the mean ES (β = 0.0271; *p* = 0.6939). All studies included in this review satisfied Criteria 1, 3, 7, and 9 through 12. None of the other individual study criteria were independently associated with the overall mean ES, which included Study Quality Criterion 2 (β = −0.1384; *p* = 0.3159), Criterion 4 (β = 0.4512; *p* = 0.1972), Criterion 5 (β = 0.4512; *p* = 0.1972), Criterion 6 (β = 0.1645; *p* = 0.4546), and Criterion 8 (β = −0.0883; *p* = 0.5011).

## 4. Discussion

The results of the current study indicate that a small–moderate decrease in vertical jump performance occurred following an exhaustive exercise bout, with the greatest change observed on Days 1 and 2 and recovery achieved by Days 3, 4, and 5. Sex, athlete status, and the type of exercise stressor were not associated with the mean ES, which indicated that force plates provided a tool sensitive enough to detect a change in vertical jump performance across various exercise stressors, and the change in performance and recovery was consistent regardless of the sex training status of the individual. Although this response was consistent for men and women, the current results are predominantly based on data collected from male participants. Interestingly, a larger average decrease was observed when measuring performance using Peak Height, which suggests this may be more sensitive than Peak Power as a measure of muscular fatigue and recovery. While it was hypothesized that athletes would recover more quickly, the average decrease in performance was similar between athletes and non-athletes; however, the time course and rate of recovery could not be thoroughly examined within each of these subgroups to determine if athletes recovered faster than non-athletes. Of the potential sources of variability examined, a larger average decrease in performance occurred in “older” participants, but the term “older” in the context of this review applied to adults in their 30s and 40s. This aligns with previous research which suggested a decrease in sport performance occurs around 35 years of age and may be due to the age-related differences in the degree of muscle damage after exercise, as well as a slower rate of repair and recovery [41,66].

While the aim of the current study was not to examine the time course of muscle soreness and biochemical markers of muscle damage, the time course of recovery observed in the current results is consistent with other research examining fatigue and recovery [67]. Muscle soreness is known to impair muscle function through mechanisms such as increased stiffness and localized swelling, potentially reducing performance. Similarly, increases in markers of muscle damage are often associated with soreness and declines in functional capacity [16]. Increased muscle soreness and markers of skeletal muscle damage typically occur within the first 24 h after a novel exercise stressor and peaks between 24 and 72 h, before eventually subsiding 5 to 7 days post-exercise [68,69]. The time course of vertical jump recovery measured using a force plate system in the current analysis aligns with the 72 h recovery window observed with other biochemical markers, such as creatine kinase and lactate dehydrogenase, which are more time consuming and more invasive to assess [70]. In contrast, other prior research found impairments in strength and power that may last up to 8 to 10 days [71]. While it may not be possible to manage fatigue and enhance recovery by avoiding exercise for this long for some athletes, the importance of adequate nutrition, passive rest, and sleep cannot be underscored [3]. In addition, untangling the relationships between chemical markers of muscle damage, perceived recovery, indices of OTS, and muscle recovery using force plate systems was beyond the scope of the current study. However, for practitioners in a clinical or performance setting, force plates present an effective, non-invasive, and user-friendly modality for monitoring recovery and their ability to provide objective data on neuromuscular function aligns well with the established 72 h recovery window, making them a valuable tool for both athletes and non-athletes.

The current results indicate Peak Height provided the most sensitive measure of performance; however, this information can be obtained using less expensive and more accessible tools (e.g., video analysis or photoelectric systems such as Optojump) while maintaining high levels of validity and reliability [72,73]. While there is no doubt regarding the accuracy and usefulness of force plates to measure Peak Height, the added value of force plates to monitor fatigue lies within their ability to provide additional metrics (e.g., FT/CT, RSImod) which may be more sensitive to neuromuscular fatigue. Many of the time and rate-related variables showed larger decreases and remained altered longer than force-based variables such as Peak Fore or Peak Power post-exercise [65]. However, these were inconsistently reported across the literature and could not be thoroughly examined in the current study. By offering insights into the recovery process through dynamic and functional assessments, force plates facilitate informed decisions about training and recovery strategies, ultimately contributing to improved performance and reduced injury risk.

It was hypothesized that athletes would recover faster than non-athletes because of their rigorous training and active lifestyle, which would likely result in muscular adaptations such as increased muscle fiber size and recruitment patterns [74], improved mitochondrial function [75], and improved blood flow [76]. However, the results of the current study found no difference in recovery based on athlete or training status. While this was implied by the athlete sub-analysis, the authors cannot confirm that every athlete was more powerful than every non-athlete in this review. However, initial jump height was also examined as a potential moderator and was not associated with the decrease in performance either. These findings underscore the complexity of recovery and suggest that further research is needed to fully understand the factors influencing recovery times across different populations, as well as to identify a more sensitive marker of recovery that captures the differences between athletes and non-athletes if one truly exists [12].

The current results indicated that Peak Height is a more sensitive measure of fatigue and recovery than Peak Power; however, this is in partial contrast to the previous literature which suggests that jump height may not be ideal to detect fatigue when used in isolation [12]. It is possible that the apparent greater sensitivity of Peak Height was influenced by characteristics of the included samples (e.g., athlete vs. non-athlete, sex, age, and exercise stressor). Most effects examined vertical jump height performance using Peak Height or Peak Power. Because the Day × Primary Outcome interaction was not associated with the observed decrease in performance, the time course of recovery measured using Peak Height was not different than when measured using Peak Power in the studies included in this review. Even though the decrease in Peak Height was larger, both measures appeared to decline and improve at relatively similar rates following an exhaustive exercise bout. Although commonly used force-based metrics showed moderate effects, novel time- and rate-related markers may offer further insight, suggesting that the recovery process could extend longer following an acute exercise stressor [65].

When analyzing Peak Height versus Peak Power in the context of a jump, it is also important to understand the nature of the data being measured. Among the 22 studies included in this review (Table 3), all but one estimated Peak Height using either the flight time method or derived metrics such as jump power from force plates [65]. However, multiple studies have emphasized that jump height estimation based on flight time may be less accurate due to factors such as knee flexion at landing, whereas the impulse–momentum method has been shown to provide greater accuracy [77,78]. The estimation of Peak Height using the impulse–momentum method via a force plate involves capturing a continuous range of data throughout the entire initial phase of the jump, up to the point of takeoff. As a result, the measurement encompasses the complete impulse generated and provides a comprehensive view of the force applied and the resulting movement over time. In contrast, Peak Power is derived from a singular timepoint during the pushing phase where force and velocity are multiplied to calculate power output. This specific moment may not fully represent the entire jumping effort, leading to a less holistic understanding of the performance. Therefore, a practitioner can observe a decrease Peak Height which often appears more drastic because it reflects the cumulative effort and dynamics of the jump, rather than a snapshot of the highest power output at a singular instant. This comprehensive data capture of the concentric impulse allows for a more detailed and accurate assessment of the jumper’s performance.

Despite the hypothesized differences in recovery between female and male participants [44,45], it is also unclear why studies with female participants measuring performance using Peak Power showed a larger decrease in vertical jump. Previous research found the decrease in Peak Height and isokinetic concentric strength was larger in female participants following an exhaustive resistance training bout [79]; however, determining whether the decrease in performance observed in the current results was due to disruptions to the muscle milieu, differences in training history, or spurious results due to characteristics of the study samples and the small number of studies with female participants warrants further research [80,81,82]. Identifying the causal factor(s) was beyond the scope of the current study and should be experimentally examined in future research.

### Strengths and Limitations

The current meta-analysis used a three-level model structure to adjust for between-study variance and the correlation between effects nested within studies. This was especially important when examining vertical jump performance using force plates using various measures across repeated days. While the magnitude of the overall change was relatively smaller than anticipated, the time course of recovery is critical and occurs along a continuum, and the magnitude and timing of the change is important to consider as even small changes in performance may be practically meaningful for coaches and athletes to gain a competitive edge and may offer a great opportunity for researchers to continue in this area.

The electronic database search identified 100% of the publications included in the final analysis, which is higher than the target sensitivity identified in previous systematic reviews [83]. Including multiple databases with a variety of possible keyword combinations is recommended as part of the systematic review strategy, and to the authors’ knowledge this approach successfully identified all relevant publications in the current review [18,51]. Although a keyword search of multiple electronic databases and manual search of references were used in the current study, it is possible that additional studies were not included in this review because they were not identified during either literature search. In addition, it is possible that additional research studies were published since the completion of the electronic database search. However, the time elapsed from the conception of this study to the search for peer-reviewed studies for inclusion, the submission of the final manuscript, and eventual publication is well within a normal time frame [84]. It is worth noting, however, that regardless of whether all research on this specific topic was included in the current review, even the conservative fail-safe estimate of over 2000 additional effects that would diminish the significance of the observed ES to a non-significant level was considered quite robust [85]. While there may be no way to truly know the number of unpublished studies that exist in the “file drawer”, it is unlikely that thousands of additional contradictory null effects exist.

Lastly, a few other limitations should be noted regarding the current body of literature. A very small number of participants were identified as female. While there were no sex-related differences in the decrease in vertical jump performance, this is likely a result of low statistical power given that the magnitude of the decrease was nearly three-fold larger among female participants. Because only one study in the current review included a combined sample of male and female participants, but did not directly compare their results, future research should examine whether the magnitude of the change in vertical jump performance following an exhaustive exercise bout is larger among females.

The authors also expected that the time course of recovery would be modified by training status, such that athletes and trained participants would recover more quickly than recreationally active or untrained participants. While not presented in the current study, results were also analyzed by stratifying participants into additional subgroups (e.g., athletes, recreationally active, untrained) and by collapsing the number of days from Day 3, Day 4, and Day 5 into a single timepoint representing “≥3 Days” to account for the relatively small number of effects which examined recovery beyond Day 3. Neither approach yielded a significant Day ×Athlete interaction. An important point to note is that the authors observed inconsistencies in how the original studies described their participants with terms like ‘athletes’, ‘trained’, or ‘recreationally active’. In some cases, participants involved in recreational endurance sports displayed aerobic capacities comparable to those of professional athletes, though they may have lacked the sport-specific skills necessary to compete at an elite level [52,62]. It still may be possible that the time course of recovery is different between athletes and non-athletes; however, these groups were not directly compared in a single study, and the current null results may be due to inconsistent definitions used to categorize “athletes”, “non-athletes”, “active”, and “inactive” by the original authors across studies. Furthermore, the authors also anticipated that the time course of recovery would also vary by the specific primary outcome reported. However, the limited number of effects which reported the change in performance for each specific primary outcome measure were limited and likely lacked statistical power to detect a difference between outcome measures and the subsequent time course of recovery for each specific outcome measure. Despite the null findings in this area, future research should examine the time course of recovery using different measures of performance to determine if one provides a more sensitive measure to detect readiness to perform.

## 5. Conclusions

Force plates offer a practical and non-invasive method for monitoring recovery in both athletes and non-athletes and can offer precise and objective data on various aspects of physical performance. Metrics such as Peak Height and Peak Power, as well as the time spent in various phases of the vertical jump, can provide clear insights into an individual’s recovery status. Vertical jump performance can be measured using force plate systems to track recovery following a strenuous exercise bout, with a decrease in performance observed on Day 1 and Day 2 of recovery before a return to baseline on Day 3. A larger decrease in performance was observed in older participants; however, the change was consistent across male and female participants and for athletes and non-athletes.

## Figures and Tables

**Table 1:** Definitions for levels of moderators.

Effector Moderator	Levels
Age	Continuous variable, the mean age of the participants reported in years
Athlete Type	Categorical variable, coded as follows:
	Athlete: data from competitive sport athletes including those involved in rugby, basketball, track and field, or soccer or other highly trained physically active participants including body builders or power athletes
	Non-athlete: data from sedentary or inactive participants
Body Mass	Continuous variable, the mean body mass of the participants reported in kg
Body Mass Index	Continuous variable, the mean body mass index of the participants reported in kg/m^2^
Day of Recovery	Continuous variable and categorical variable, coded with values ranging from 1 to 7 days
Exercise Stressor	Categorical variable, coded as follows:
	Game: data collected following in-game or simulated game competition
	Laboratory-based: data collected following high-intensity resistance or aerobic exercise protocol
Height	Continuous variable, the mean height of the participants reported in cm
Percent Female	Continuous variable, the percentage (0–100) of the experimental group self-reporting female gender
Sex Group	Categorical variable, coded as follows:
	Female: data from female participants only
	Male: data from males participants only
	Mixed: data from combined samples of female and male participants
Year Published	Continuous variable, year of publication

**Table 2:** Participant characteristics from studies examining the change in vertical jump performance using force plates.

	N	Mean	SD	Min	Max
Age (yrs)	286	24.70	4.57	18.00	45.00
Body Mass (kg)	278	78.53	7.71	59.80	93.10
BMI (kg/m^2^)	284	24.36	1.77	21.10	28.15
Height (cm)	266	179.00	4.57	165.90	187.60

Note: BMI, body mass index. cm, centimeters. kg, kilogram. kg/m^2^, weight in kilograms expressed relative to height measured in meters squared. min, minimum. max, maximum. N, total number of participants. SD, standard deviation.

**Table 3:** Description of studies examining the change in vertical jump performance using force plates.

Study Name	Participant Description	Age	Height	Body Mass	Study Overview and Investigated Outcomes
Aben et al., 2020 [26]	Male Rugby League Players (n = 10)	18 ± 1 yrs	183 ± 4 cm	92 ± 9 kg	Countermovement Jump Height and Velocity up to 120 h following rugby league match play
Boullosa et al., 2021 [52]	Physically Active Men (n = 12)	23.4 ± 2.8 yrs	183 ± 6 cm	76.6 ± 7.8 kg	Countermovement Jump Height, Peak Power, and Vertical Stiffness up to 24 h following concentric and eccentric cycling
Cochrane et al., 2013 [53]	Physically Active Men (n = 10)	21.0 ± 1.7 yrs	178.6 ± 6.5 cm	79.2 ± 12.8 kg	Countermovement Jump Height and Peak Power up to 72 h following 3 sets of 100 maximum eccentric contractions (quadriceps)
Collins et al., 2019 [54]	Male Sport Athletes (n = 10)	23.0 ± 4.5 yrs	182.1 ± 9.5 cm	82.3 ± 12.9 kg	Countermovement Jump Height, Relative Peak Power, and the ratio between Flight Time and Contact Time up to 24 h following high-intensity sprint and plyometric exercise
Dourado et al., 2022 [55]	Untrained Young Men (n = 14)	22.8 ± 3.6 yrs	175 ± 9 cm	79.1 ± 9.7 kg	Countermovement Jump Height, Peak Power, and Vertical Stiffness up to 96 h following single- and multi-joint lower body resistance exercise
Fonda et al., 2015 [56]	Healthy Female Adults (n = 12)	21.0 ± 2.1 yrs	168.5 ± 4.5 cm	59.9 ± 8.5 kg	Countermovement Jump Height, Force, Power, and Energy up to 96 h following 50 drop jumps and 50 hamstring leg curls
Hotfiel et al., 2021 [57]	Healthy Adults (n = 8 women, 10 men)	24.1 ± 3.6 yrs	NR	NR	Countermovement Jump Height, Force, Power, and Energy up to 48 h following 50 drop jumps and lower body eccentric exercises
Kirby et al., 2012 [58]	Untrained Males (n = 8)	21.1 ± 1.7 yrs	178.7 ± 4.1 cm	76.7 ± 14.8 kg	Countermovement Jump Height up to 96 h following 100 drop jumps and eccentric leg press exercise
Kotikangas et al., 2022 [27]	Power Athletes (n = 8)	23.9 ± 2.9 yrs 24.1 ± 2.7 yrs 27.6 ± 2.8 yrs	181.1 ± 3.6 cm	80.5 ± 8.4 kg	Countermovement Jump Height up to 48 h following power, strength, and hypertrophic loading
	Strength Athletes (n = 8)		176.6 ± 6.3 cm	83.2 ± 13.0 kg	
	Non-Athletes (n = 7)		186.6 ± 7.2 cm	93.1 ± 17.4 kg	
Kraemer et al., 2007 [28]	Resistance-Trained Men (n = 9)	22 ± 3 yrs	179 ± 8 cm	89.7 ± 14.9 kg	Countermovement Jump Power up to 48 h following lower body resistance exercise
Levitt et al., 2020 [59]	Resistance-Trained Men (n = 10)	21–28 yrs (mean and SD not reported)	179.5 ± 5.6 cm	85.9 ± 13.2 kg	Countermovement Jump Height, Power, and Force up to 48 h following lower body eccentric resistance exercise
Li et al., 2023 [60]	Amateur Male Basketball Players (n = 10)	22.8 ± 0.8 yrs	179 ± 4 cm	75.6 ± 6.6 kg	Countermovement Jump Height up to 24 h following a simulated load basketball game
Prowting et al., 2021 [61]	Resistance-Trained Men (n = 8)	22.3 ± 2.5 yrs	175.9 ± 8.3 cm	87.1 ± 19.1 kg	Countermovement Jump Height up to 120 h following 100 drop jumps
Russell et al., 2015 [62]	Professional Soccer Players (n = 5)	21 ± 1 yrs	177.0 ± 3.0 cm	70.4 ± 2.3 kg	Countermovement Jump Height and Peak Power up to 48 h following soccer match play
Schumann et al., 2013 [29]	Untrained Young Men (n = 42)	29.2 ± 4.9 yrs	178.3 ± 5.2 cm	75.9 ± 8.6 kg	Countermovement Jump Height up to 48 h following combined strength and endurance loading
Skurvydas et al., 2000 [30]	Healthy Untrained Men (n = 12)	25.4 ± 1.7 yrs	NR	74.3 ± 6.2 kg	Squat and Countermovement Jump Height up to 24 h following 100 drop jumps
Skurvydas et al., 2006 [31]	Healthy Untrained Men (n = 20)	20.4 ± 1.7 yrs	180.7 ± 6.5 cm	76.2 ± 4.7 kg	Drop Jump Height up to 72 h following 100 drop jumps
Taipale et al., 2014 [33]	Recreationally Trained Women (n = 10) and Men (n = 12)	33.5 ± 8.3 yrs 38.8 ± 7.1 yrs	165.9 ± 7.6 cm	59.8 ± 5.1 kg	Countermovement Jump Height up to 48 h following combined strength and endurance loading
			177.4 ± 6.4 cm	75.7 ± 3.6 kg	
Taipale et al., 2018 [32]	Physically Active Men (n = 11)	25.7 ± 3.9 yrs	181.0 ± 7.7 cm	78.4 ± 10.9 kg	Countermovement Jump Height up to 19 h following lower body resistance loading
West et al., 2014 [63]	Elite International Rugby Players (n = 10)	26 ± 5 yrs	183 ± 8 cm	86.1 ± 10.0 kg	Countermovement Jump Height and Peak Power up to 48 h following rugby tournament competition
White et al., 2014 [64]	Healthy Recreationally Active Men (n = 8)	23.6 ± 3.7 yrs	180.8 ± 8.1 cm	76.1 ± 8.6 kg	Squat and Drop Jump Height and Peak Power up to 48 h following high-intensity sprint exercise
Yoshida et al., 2023 [65]	Male College Basketball Players (n = 11)	19.9 ± 1.0 yrs	187.6 ± 13.7 cm	88.4 ± 12.2 kg	Countermovement Jump Height, Net Impulse, Relative Net Impulse, Relative Peak Force, Relative Mean Force, Relative Mean Power, Total Time to Peak Force, Total Time to Peak Power, Average Rate of Force Development, and Ratio of Flight Time to Contraction Time up to 72 h following basketball-related high intensity exercises

Note: cm, centimeters. kg, kilograms. NR, not reported. yrs, years.

**Table 4:** Summary of univariate moderator analysis from studies examining the change in vertical jump performance using force plates.

	β	95%CI	*p*
Age (yrs)	−0.0489	−0.0719 to −0.0258	<0.0001
Body Mass (kg)	0.0014	−0.0112 to 0.0140	0.8323
BMI (kg/m^2^)	−0.0166	−0.0767 to 0.0434	0.5848
Days of Recovery	0.1370	0.0875 to 0.1864	<0.0001
Height (cm)	0.0045	−0.0135 to 0.0225	0.6240
Percent Female	0.0875	−0.2960 to 0.4710	0.6531
Publication Year	−0.0003	−0.0208 to 0.0203	0.9800

Note: BMI, body mass index. cm, centimeters. kg, kilograms. kg/m^2^, body mass in kilograms expressed relative to height in meters squared. yrs, years. β, reported as unstandardized beta coefficients.

**Table 5:** Summary of subgroup moderator analysis from studies examining the change in vertical jump performance using force plates.

	*k*	ES	95% CI	Within *p*	Between *p*
Athlete Status					
Athlete	101	−0.1651	−0.3122 −0.0179	0.0283	
Non-Athlete	83	−0.3285	−0.5111 −0.145	0.0006	0.4476
					
Exercise Stressor					
In-Game or Simulated Game Setting	70	−0.3299	−0.6585 −0.0012	0.0492	
Laboratory Setting	114	−0.2751	−0.4170 −0.1332	0.0002	0.5803
					
Primary Outcome					
Peak Height	88	−0.4687	−0.7635 −0.1732	0.0022	
Peak Power	23	−0.1399	−0.3002 0.0203	0.0838	0.0393
					
Sex					
Female	16	−0.9528	−1.7507 −0.1550	0.0224	
Male	167	−0.2747	−0.4128 −0.1365	0.0001	0.6531

Note: *k*, number of effects. ES, effect size. CI, confidence interval.

**Table 6:** Study quality characteristics of studies examining the change in vertical jump performance using force plates.

	NIH Scale Question Number												
Study Name	1	2	3	4	5	6	7	8	9	10	11	12	Total Score
Aben et al., 2020 [26]	Y	N	Y	Y	Y	N	Y	N	Y	Y	Y	Y	9
Boullosa et al., 2021 [52]	Y	Y	Y	Y	Y	Y	Y	N	Y	Y	Y	Y	11
Cochrane et al., 2013 [53]	Y	N	Y	Y	Y	Y	Y	N	Y	Y	Y	Y	10
Collins et al., 2019 [54]	Y	Y	Y	Y	Y	Y	Y	N	Y	Y	Y	Y	11
Dourado et al., 2023 [55]	Y	Y	Y	Y	Y	Y	Y	N	Y	Y	Y	Y	11
Fonda et al., 2015 [56]	Y	Y	Y	Y	Y	Y	Y	N	Y	Y	Y	Y	11
Hotfiel et al., 2021 [57]	Y	Y	Y	Y	Y	Y	Y	N	Y	Y	Y	Y	11
Kirby et al., 2012 [58]	Y	Y	Y	Y	Y	Y	Y	Y	Y	Y	Y	Y	12
Kotikangas et al., 2022 [27]	Y	Y	Y	Y	Y	Y	Y	N	Y	Y	Y	Y	11
Kraemer et al., 2007 [28]	Y	Y	Y	Y	Y	Y	Y	N	Y	Y	Y	Y	11
Levitt et al., 2020 [59]	Y	N	Y	Y	Y	Y	Y	N	Y	Y	Y	Y	10
Li et al., 2023 [60]	Y	Y	Y	Y	Y	Y	Y	N	Y	Y	Y	Y	11
Prowting et al., 2021 [61]	Y	Y	Y	Y	Y	Y	Y	Y	Y	Y	Y	Y	12
Russell et al., 2015 [62]	Y	Y	Y	N	N	N	Y	N	Y	Y	Y	Y	8
Schumann et al., 2013 [29]	Y	N	Y	Y	Y	Y	Y	N	Y	Y	Y	Y	10
Skurvydas et al., 2000 [30]	Y	Y	Y	Y	Y	Y	Y	N	Y	Y	Y	Y	11
Skurvydas et al., 2006 [31]	Y	Y	Y	Y	Y	Y	Y	N	Y	Y	Y	Y	11
Taipale et al., 2014 [33]	Y	Y	Y	Y	Y	Y	Y	N	Y	Y	Y	Y	11
Taipale et al., 2018 [32]	Y	Y	Y	Y	Y	Y	Y	N	Y	Y	Y	Y	11
West et al., 2014 [63]	Y	N	Y	Y	Y	Y	Y	N	Y	Y	Y	Y	10
White et al., 2014 [64]	Y	Y	Y	Y	Y	Y	Y	N	Y	Y	Y	Y	11
Yoshida et al., 2023 [65]	Y	N	Y	Y	Y	Y	Y	N	Y	Y	Y	Y	10

Note: NIH Quality Assessment Tool for Before–After (Pre–Post) Studies with No Control Group. Y, yes. N, no.

## Data Availability

Data used for these analyses are available in a public repository that is accessible through the University of Alabama, which does not issue datasets with DOIs (non-mandated deposition). The data can be downloaded directly from https://ir.ua.edu/handle/123456789/2871 in Microsoft Excel file format.

## Supplementary Materials

The following supporting information can be downloaded at https://www.mdpi.com/article/10.3390/jfmk10020230/s1.
